# Infection curves on small-world networks are linear only in the vicinity of the critical point

**DOI:** 10.1073/pnas.2024297118

**Published:** 2021-02-26

**Authors:** Łukasz Kuśmierz, Taro Toyoizumi

**Affiliations:** ^a^Laboratory for Neural Computation and Adaptation, RIKEN Center for Brain Science, Saitama 351-0198, Japan;; ^b^Department of Mathematical Informatics, Graduate School of Information Science and Technology, The University of Tokyo, Tokyo 113-8656, Japan

In ref. 1, an epidemiological model based on the small-world (2) contact network and the SIR (susceptible-infected-recovered) infection dynamics is analyzed and compared with the classical (well-mixed) SIR model. The authors claim that they observe a “a hitherto unobserved transition from linear growth to S-shaped infection curves.” This is rather surprising in the context of previous literature studying similar models (3–9). While scale-free networks (10) were reported to give rise to nonstandard features, including the absence of a nontrivial epidemic threshold and an algebraic spreading growth (6–8), no such abnormalities were observed on small-world networks (3–5, 7).

Indeed, in the following, we show that the analysis presented in the paper (1) is flawed. The problem lies in the order parameter O=SD(C(t)), the standard deviation of new daily cases (excluding days with no new cases), which is used to signal the postulated transition between an S-shaped growth (O>0) and a linear growth (O≈0). In reality, O can attain values close to zero for two distinct reasons: 1) C(t) is constant (corresponding to a linear growth of the cumulative number of cases) as the authors claim, or 2) C(t) exhibits low values, signaling that the epidemic is not able to effectively spread in the population and the number of cases drops exponentially, corresponding to the basic reproduction number $R_{0}$ taking a value below one. Case 2, which is in line with the prediction of the classical SIR model, was never considered in ref. 1; both theoretical and numerical calculations established the existence of a critical point, but no evidence was reported for the existence of the postulated linear growth phase below the critical point.

**Fig. 1.**
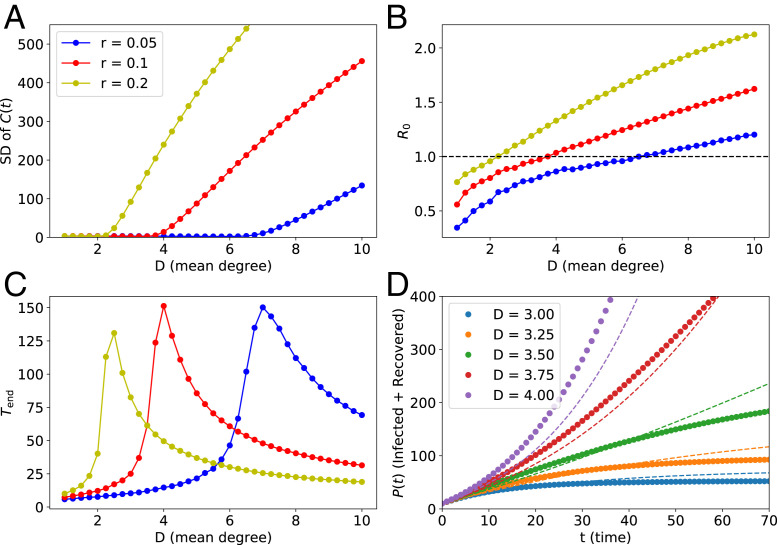
Statistics obtained via computer simulations of the epidemic spread on Poisson small-world random contact networks, defined in ref. 1. Parameters are the same as in figure 3*A* in ref. 1 (d=4, ϵ=0.3, and $N=10^{4}$). The simulation starts from 10 randomly infected nodes. All statistics were obtained by averaging over $M=10^{3}$ realizations of the process, each with an independent realization of the random graph. (*A*) The SD of daily cases; this panel confirms that we reproduce results from ref. 1 (see their figure 3*A*). In ref. 1, the low SD(C(t)) regions are interpreted as linear growth regimes. (*B*) The basic reproduction number as a function of the average degree of the network. The dashed line represents the critical value $R_{0}=1$. Below the critical point, the outbreak dies out exponentially; our numerical estimates confirm $R_{0}<1$ in this regime. (*C*) The outbreak duration $T_{\text{end}}$, that is, the average time elapsed between the onset of an epidemic and its end (defined as the first time at which there are no infected nodes in the population). We observe a sharp peak around the critical point, confirming that the slow dynamics, consistent with a linear growth, is only possible around the critical point. (*D*) The cumulative numbers of infections as a function of time (here r=0.1). The dashed lines in *D* were obtained from the corresponding effective classical SIR models, which assume continuous time and homogeneous mixing. Although the match is not perfect, the classical model can reproduce qualitative features of the network model studied in ref. 1.

We hypothesized that case 2 is the mechanism yielding O≈0 below the critical point. To test this, we independently simulated the model introduced in ref. 1. First, we verified that O signals a continuous transition between two distinct phases (Fig. 1*A*). Second, we calculated the basic reproduction number $R_{0}$ (Fig. 1*B*), which confirmed that, below the critical point, an infection does not spread through the network, consistent with case 2. Third, we calculated the outbreak duration $T_{\text{end}}$, which exhibits a sharp maximum at the critical point and drops especially quickly below the critical point (Fig. 1*C*). Because $T_{\text{end}}$ upper-bounds the possible duration of a linear growth, this result indicates that prolonged linear growth is impossible even slightly below the critical point. Last, we compared the evolution of the cumulative number of infections obtained from the small-world model and the classical SIR model (Fig. 1*D*). Already slightly below the critical point, the growth curve of the network model is significantly sublinear, as expected from the classical picture.

**Fig. 2.**
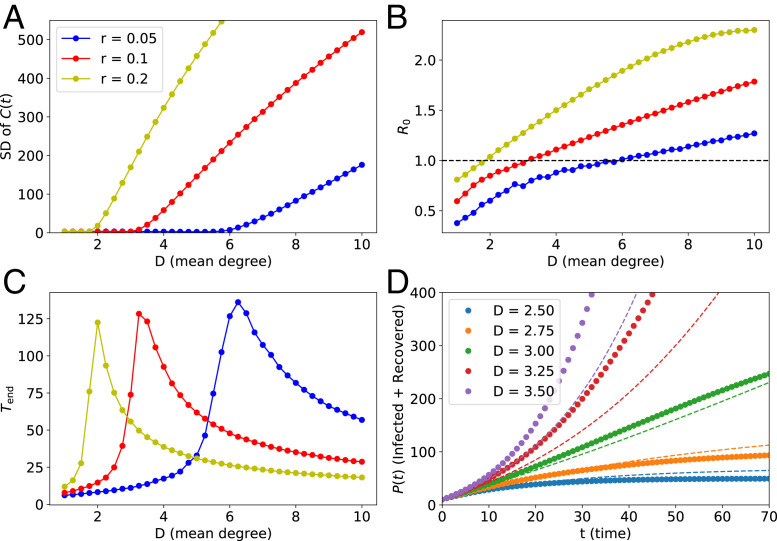
Statistics obtained via computer simulations of the epidemic spread on Erdős–Rényi contact networks. The meaning of *A*–*D* and parameters used in the simulations are the same as in Fig. 1 (excluding the parameter ε, which does not apply here).

In conclusion, the presented results corroborate our supposition that the linear growth in the cumulative number of infections is restricted to the vicinity of the critical point. Furthermore, we checked that the results are very similar on Erdős–Rényi networks (Fig. 2). Thus, the model introduced in ref. 1 does not predict that nor explain why “most COVID-19 infection curves are linear.”
